# A Rare Presentation of Swyer-James-Macleod Syndrome in the Elderly

**DOI:** 10.7759/cureus.70010

**Published:** 2024-09-23

**Authors:** Malavika P Premnath, Abdul Majeed Arshad, Irfan Ismail Ayub, Thangaswamy Dhanasekar

**Affiliations:** 1 Pulmonology, Sri Ramachandra Institute of Higher Education and Research, Chennai, IND; 2 Pulmonary and Critical Care Medicine, Sri Ramachandra Institute of Higher Education and Research, Chennai, IND

**Keywords:** bronchiectasis exacerbation, bronchiolitis obliterans, hyperlucent lung syndrome, pulmonary function test, swyer-james-macleod syndrome

## Abstract

Swyer-James-MacLeod syndrome (SJMS) also known as hyperlucent lung syndrome is a condition that occurs as a complication of infectious bronchiolitis obliterans. It is characterized by inflammation and fibrosis of the affected area of the lung resulting in ventilation and perfusion mismatch ultimately leading to underdevelopment of the affected lung. A key feature used for diagnosis is unilateral small lung with hyperlucency on a chest radiograph. Additional insights can be gained through high-resolution computed tomography scans. This study focuses on detailing the imaging findings from a case involving an elderly patient diagnosed with SJMS.

## Introduction

Swyer-James-MacLeod syndrome (SJMS) was initially documented by Canadian radiologist George James and pulmonologist William Mathieson Macleod in 1953, with further contributions made by Canadian physician Paul Robert Swyer in 1954. It is commonly observed as a secondary condition that follows childhood bronchiolitis obliterans. The diagnosis is established based on distinctive imaging results. This syndrome is distinguished by the radiographic hallmarks of a hyperlucent appearance in either a single pulmonary lobe or the entire lung, along with a reduction in lung volume [1]. Pulmonary function testing commonly indicates the presence of air-flow obstruction, while ventilation and perfusion scanning frequently show a significant decrease in perfusion in the affected lung. Usually, this condition is identified during childhood after evaluating infections. However, individuals who experience fewer or no complications of bronchiectasis may exhibit minor symptoms or be asymptomatic, resulting in a potential late diagnosis in adulthood [2]. 

## Case presentation

A 62-year-old male patient known case of systemic hypertension and dyslipidemia presented with a history of moderate hemoptysis for three days. He had a documented history of recurring respiratory tract infections since the age of eight. He had a history of multiple admissions during childhood for recurrent infections and was managed with antibiotics and a bronchodilator. He denied tobacco, alcohol, and recreational drug use. His family history was noncontributory. Upon examination, it was observed that he maintained hemodynamic stability, with a blood pressure of 112/76 mm Hg, a pulse rate of 82/min, a respiratory rate of 16/min, and a saturation level of 98% on room air. During the examination of the lower respiratory tract, there was a noticeable reduction in breath sounds at the base of the left lung, accompanied by crepitations. The laboratory analysis showed a hemoglobin level of 8.6g/dL, a white cell count of 10,700/mm^3^, and a platelet count of 2,55,000/mm^3^, and all liver function, renal function, and serum electrolyte tests returned within normal ranges. Serology markers were negative. Table 1 shows the laboratory investigations of the patient.

**Table 1:** Laboratory investigations of the patient.

Laboratory Parameters	Patient Value	Reference Range
Hemogram		
Hemoglobin	8.6 g/dL	12-15 g/dL
Mean Corpuscular Volume	59.4 fL	83-101 fL
White Blood Cell Count	10.7 ×10^3^/μL	4-11 ×10^3^/μL
Platelets	255 ×10^3^/μL	150-450 ×10^3^/μL
Erythrocyte Sedimentation Rate	12 mm/hour	<37.5 mm/hour,
HbA_1_c	5.20%	< 5.6%
Renal Function Tests		
Blood Urea Nitrogen	10 mg/dL	7.9-20.1 mg/dL
Creatinine	0.6 mg/dL	0.6-1.2 mg/dL
Sodium	136 mmol/L	136-146 mmol/L
Potassium	4.6 mmol/L	3.5-5.1 mmol/L
Chloride	91 mmol/L	101-109
Bicarbonate	28 mmol/L	21-27 mmol/L
Liver Function Tests		
Aspartate Aminotransferase	22 IU/L	<35 IU/L
Alanine Aminotransferase	32 IU/L	<35 IU/L
Alkaline Phosphatase	54 IU/L	30-120 IU/L
Albumin	4.5 g/dL	3.5-5.2 g/dL
Globulin	1.8 g/dL	2-3.5 g/dL
Total Bilirubin	1.2 mg/dL	0.3-1.2 mg/dL
Blood Gas Analysis		
PH	7.37	7.350-7.450
PCO_2_	39 mmHg	32 – 48 mmHg
PO_2_	82 mmHg	83- 108 mmHg
cHCO_3_	24 mmol/L	-
Lactate	0.8 mmol/L	0.20 – 1.80 mmol/L

**Figure 1:**
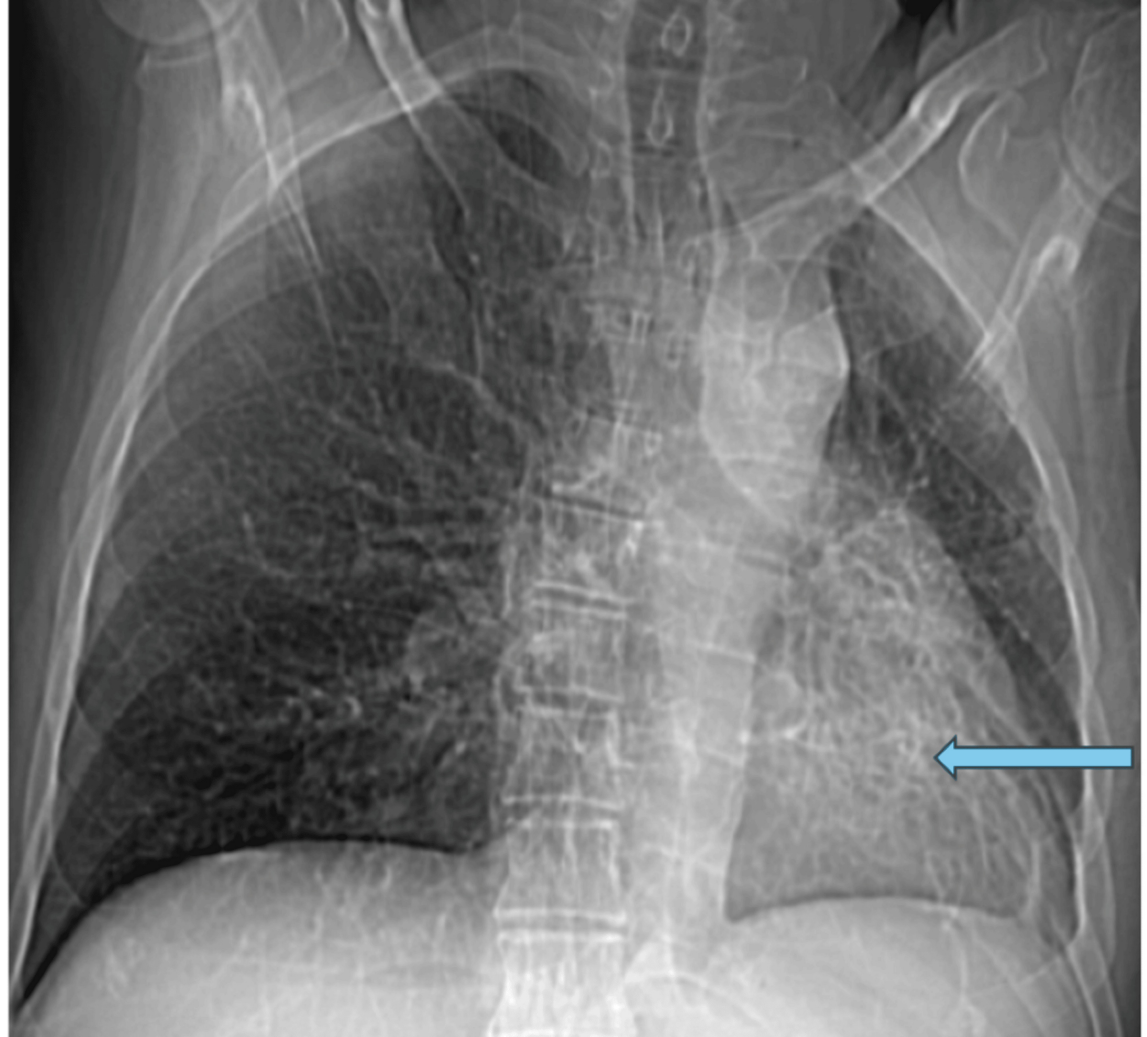
CT scout films show unilateral hyperlucency with small left lung and cystic bronchiectasis in the left lower zone lung fields.

The initial chest radiograph revealed a leftward shift of the trachea, a hyperlucent left lung with reduced volume, and a cystic change in the lower zone. Similar observations were made in the CT film as illustrated in Figure 1.

**Figure 2:**
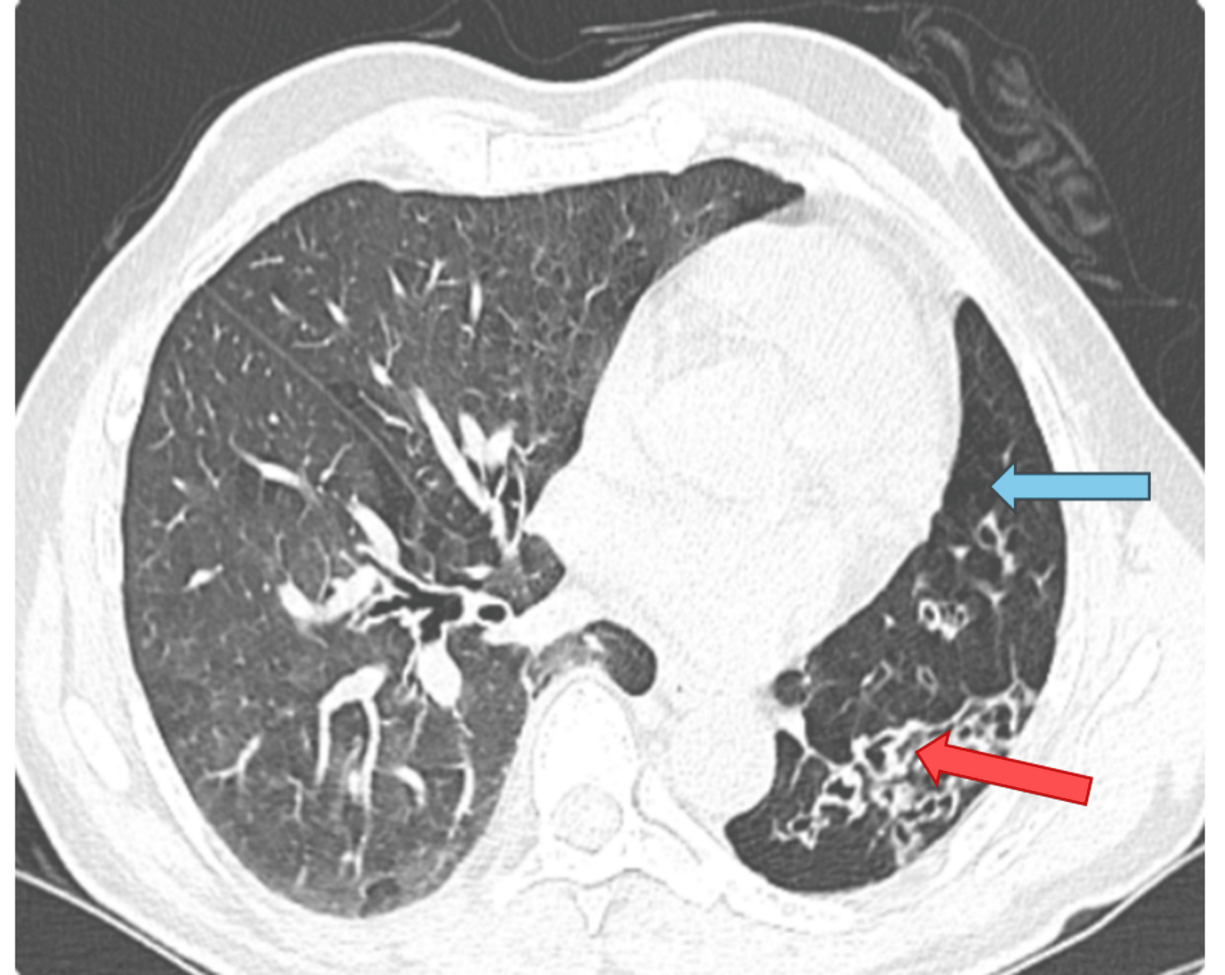
Axial-section CECT chest (lung window) shows diffuse hypoattenuation (blue arrow) of the left lung with left lower lobe cystic bronchiectasis (red arrow). CECT: Contrast-enhanced computed tomography

**Figure 3:**
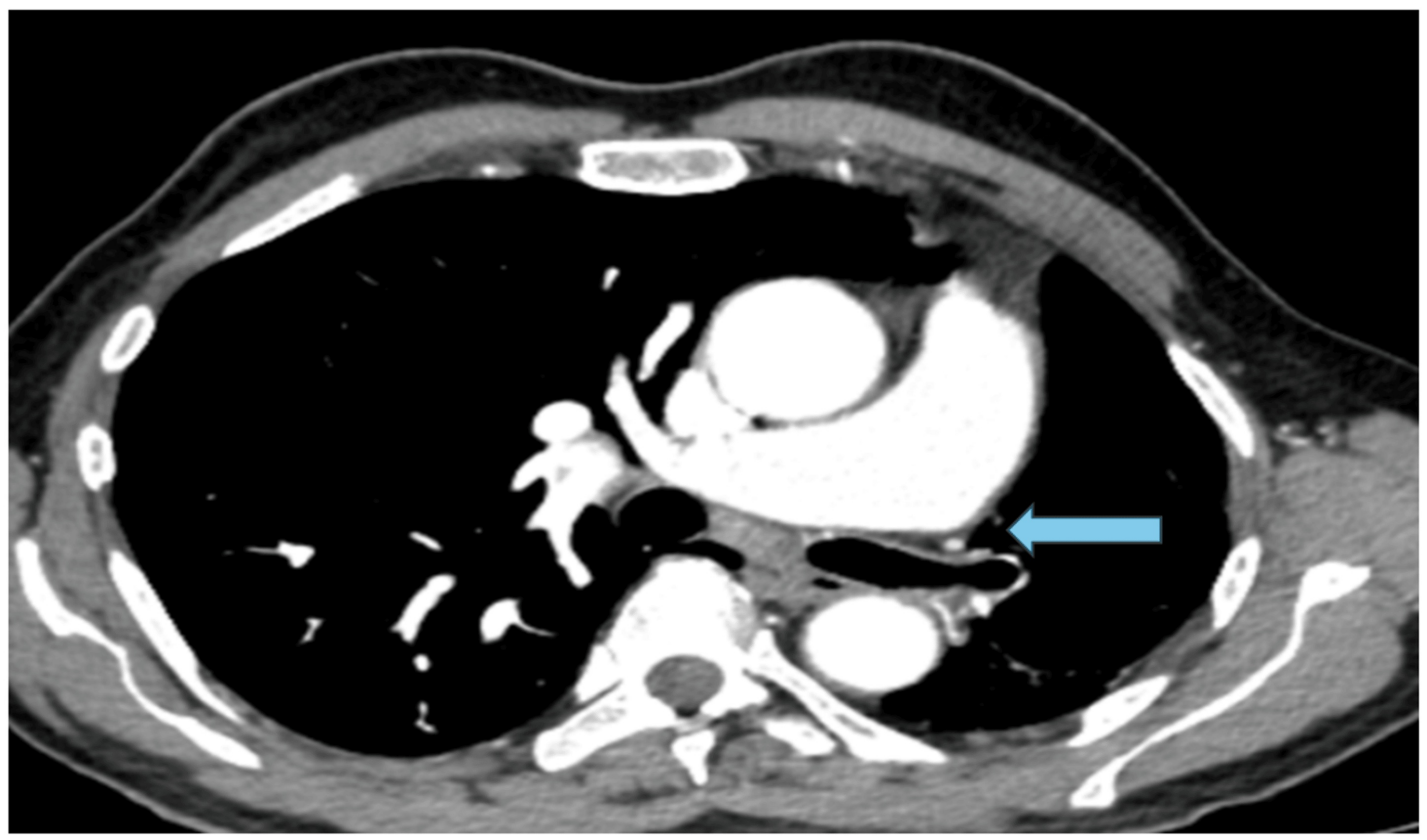
Axial CECT chest (mediastinal window) shows reduced caliber of the left pulmonary artery which was hypoplastic. CECT: Contrast-enhanced computed tomography

**Figure 4:**
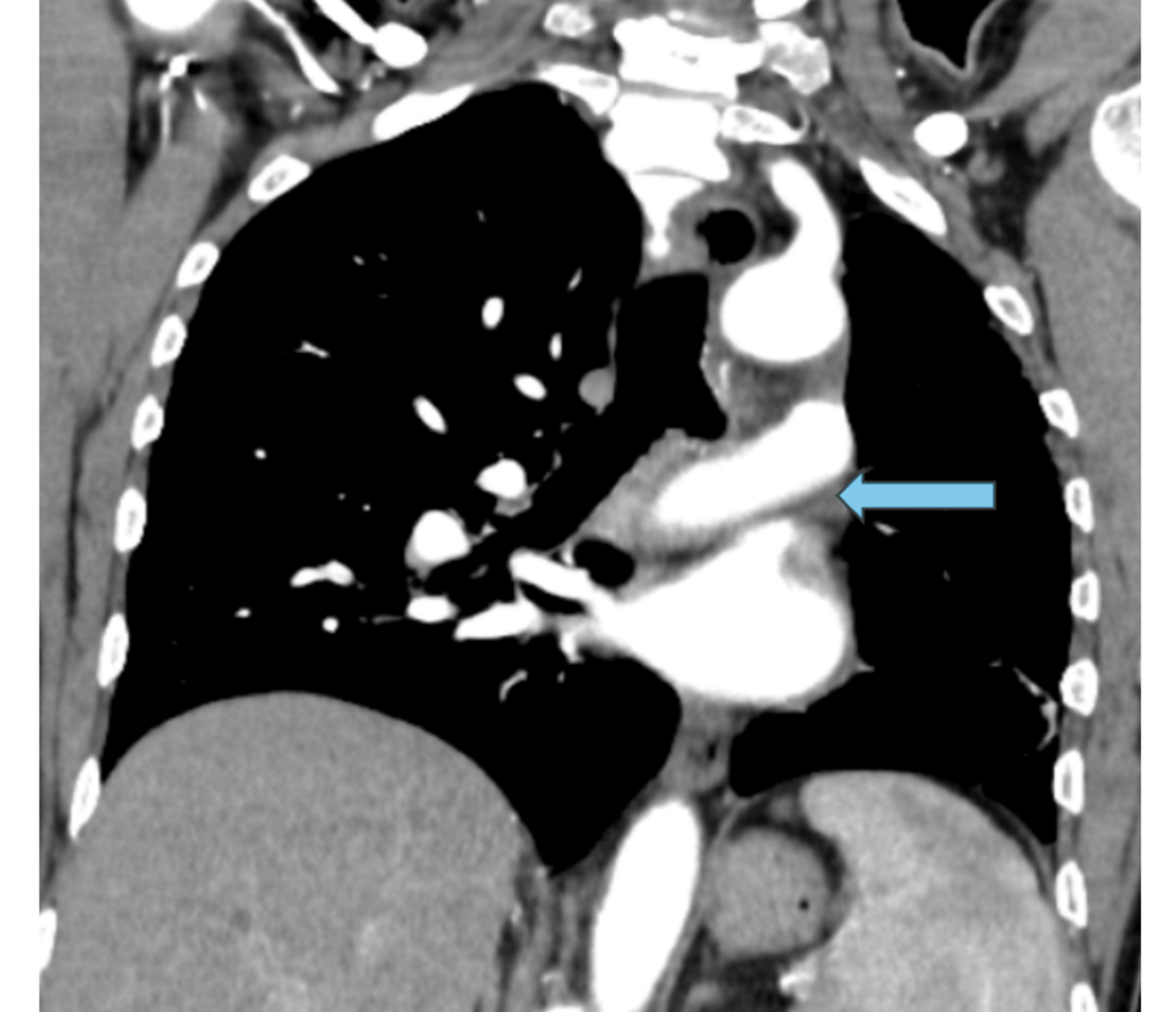
Coronal MIP shows a decreased caliber of the left main pulmonary artery (blue arrow) and its lobar and segmental branches. MIP: Maximum intensity projection

**Figure 5:**
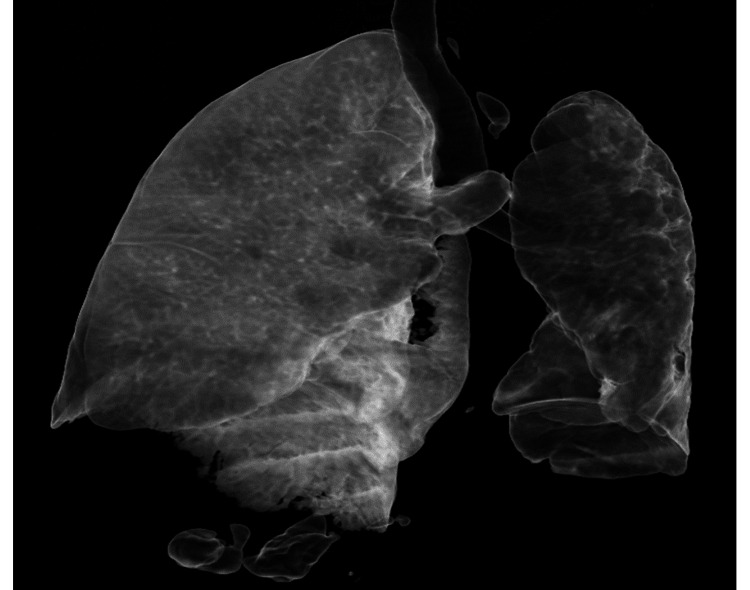
CECT chest 3D reconstruction image shows unilateral diffuse oligemia in the left lung. CECT: Contrast-enhanced computed tomography

The contrast-enhanced computed tomography (CECT) scan of the thorax, both plain and with contrast, revealed heightened translucency and diminished vascularity in the left lung, along with compensatory hyperinflation of the right lung. Additionally, thickening of the bronchial wall and the presence of cystic bronchiectasis in the lower lobes and lingula of the left lung were observed. There was mild dilation observed in the main pulmonary trunk and the right main pulmonary artery. The left pulmonary artery appeared hypoplastic and there was a generalized reduction in vascular channels throughout the left lung. The left bronchial artery displayed a mild degree of dilation and tortuosity (Figures 2-5).

**Figure 6:**
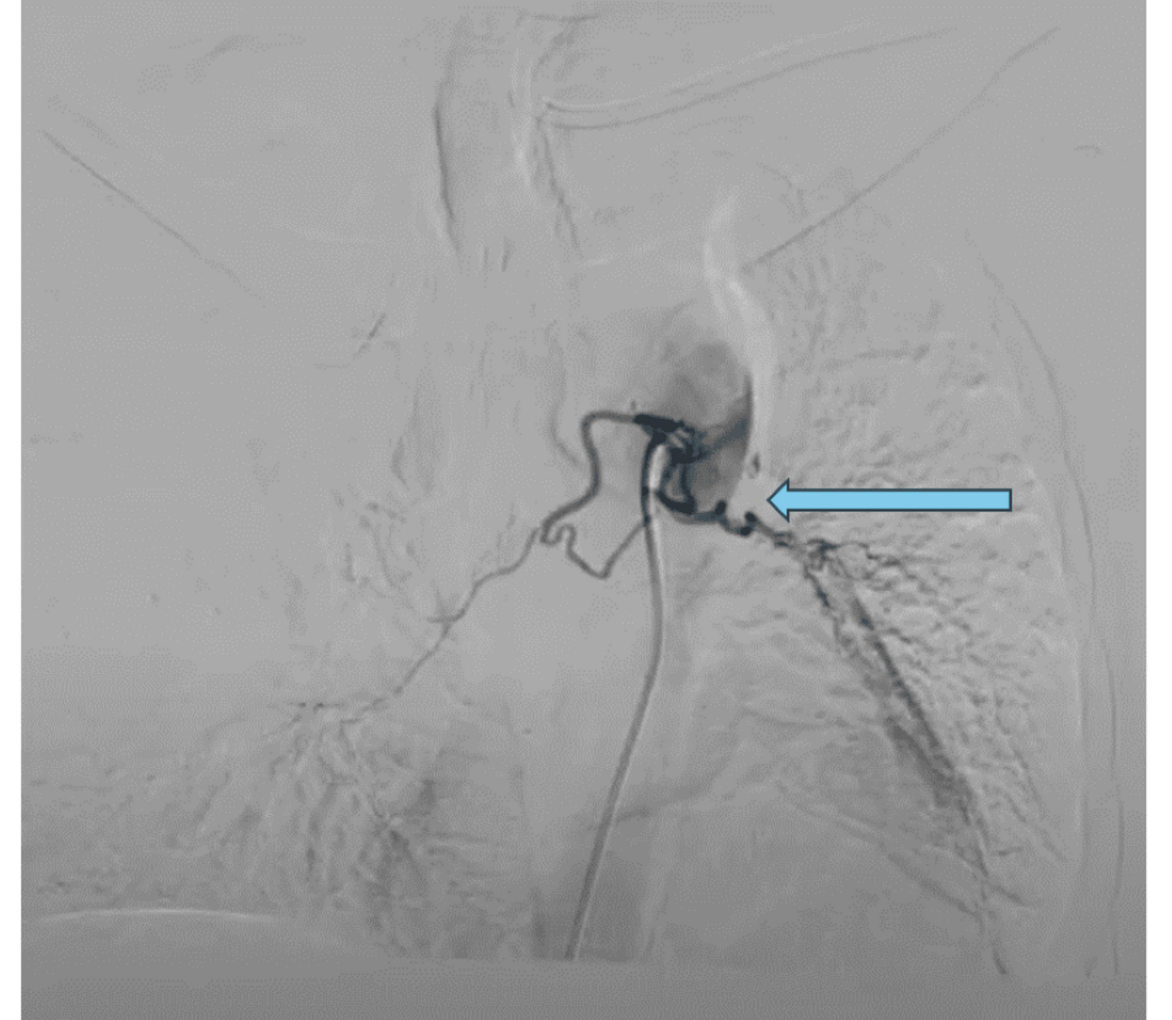
Selective catheter angiography shows cannulation of bilateral bronchial arteries at the D5-6 level with the left bronchial artery showing abnormal vascular blush.

2D ECHO revealed normal chamber dimensions, normal pulmonary artery pressure, absence of regional wall motion abnormalities, and a satisfactory ejection fraction. The treatment strategy was devised to target effectively the existing symptoms, as well as the underlying pathology. Intervention radiologists were engaged, and the patient successfully underwent endovascular embolization of the left bronchial artery at the D5 and D6 level as shown in Figure 6. The post-procedure period was free of any complications.

To address the suspected bronchial infection, a prescription for intravenous broad-spectrum antibiotics was given, along with a combination of long-acting beta-agonist and inhaled corticosteroids to alleviate bronchial inflammation. Simultaneously, a specific pulmonary rehabilitation program was initiated, beginning with controlled exercise sessions that gradually intensified, focusing primarily on improving lung capacity and strengthening respiratory muscles. 

On the day of discharge, the patient received pneumococcal and influenza vaccines. During the follow-up, both the patient's exercise tolerance and quality of life improved, and the hemoptysis resolved.

## Discussion

SJMS is a lung condition that occurs following post-infectious bronchiolitis obliterans. The first recorded case of SJMS was described by Swyer James in a six-year-old child in 1953 followed by Macleod reporting the syndrome in adults for the time, in 1954 [3]. Studies have indicated a prevalence rate of 0.01% for SJMS with cases detected in children and only a small number diagnosed in adults [4]. It has been observed that the syndrome typically develops after a lung infection during childhood often triggered by agents, like adenovirus, measles, paramyxovirus, Bordetella pertussis, tuberculosis, and Mycoplasma pneumoniae [5].

The presence of these organisms can lead to bronchiolitis, a condition marked by the narrowing of the small airways and the emergence of a severe emphysematous pattern that causes the destruction of the alveoli and dilation of the lung parenchyma. The existence of inflammation leads to a decrease in peripheral pulmonary vascularization.

These pathophysiological changes cause hyperlucency in the involved lobe, leading to air trapping and hypoperfusion in the affected segment [6]. Clinically symptomatic patients may present with productive cough, hemoptysis, recurrent pulmonary infection, dyspnea on exertion, and decreased exercise tolerance, as seen in this case. Bronchiectasis was present in 30% of the cases [7]. The diagnosis was confirmed through radiographic assessment involving X-rays and CT scans, and in certain scenarios, it was an unexpected finding. The chest CT scan of SJMS shows a region of hyperlucency resulting from diminished pulmonary perfusion, hypoplastic left pulmonary artery, and reduced unilateral vascularity, causing volume loss. CT scan findings may also include bronchiectasis, bronchiolectasis, atelectasis, and scarring [8]. This patient has a history of multiple admissions during childhood due to a recurrent infection, which was managed with antibiotics and bronchodilators. The current admission was justified by the clinical suspicion stemming from a childhood recurrent infection with moderate hemoptysis. A chest X-ray revealed hyperlucency in the left small lung. A CT pulmonary angiogram showed features suggestive of Swyer-James syndrome.

Including pulmonary angiography as a diagnostic criterion for the entity is not essential, but it has the potential to uncover hypoplasia and the reduced size of the affected pulmonary artery. Pulmonary angiography possesses inherent limitations, particularly in differentiating between congenital and acquired etiologies of hypoplastic pulmonary vasculature [9].

When assessing someone, with one lung transparency it is important to consider various potential causes like collapsed lung unevenly distributed emphysema, congenital lobe overexpansion, and underdeveloped pulmonary artery [10].

Management approaches can vary from care to surgery. Typically managing symptoms conservatively is the treatment for individuals diagnosed with SJMS by techniques like chest physiotherapy and using bronchodilators alongside getting vaccinated for pneumococcal and influenza [11,12].

Patients who demonstrate persistent chest infections and recurrent hemoptysis, despite receiving optimal medical management, should be evaluated for potential surgical intervention. The goal of the surgical intervention is to improve symptoms and improve pulmonary functions. Various surgical therapies, including segmentectomy, lobectomy with thoracotomy or video-assisted thoracoscopic surgery, and pneumonectomy with thoracotomy, have been documented [13].

Nonspecific respiratory symptoms often unmask the underlying condition. The affected lung may not be able to effectively participate in gas exchange due to hypoplasia of the airways and blood vessels, resulting in a ventilation perfusion mismatch. The comprehensive assessment underscores the significance of radiological imaging, particularly the effects of high-resolution computer tomography on bronchiectasis. Some individuals may have relatively stable lung function with appropriate management, while others may experience progressive respiratory issues. The goal of surgical intervention is to improve symptoms and pulmonary functions. Documentation includes various surgical therapies such as segmentectomy, lobectomy with thoracotomy or video-assisted thoracoscopic surgery, and pneumonectomy with thoracotomy.

## Conclusions

SJMS is an uncommon cause of unilateral hyperlucent hemithorax. This condition is commonly diagnosed during childhood when the child displays recurring respiratory infections. Nevertheless, there are a few cases where adult patients may present with the disease either incidentally or because of delayed symptom manifestation. HRCT thorax is crucial for diagnosing SJMS in adults, and consultation with a pulmonologist in a tertiary care setting is vital for comprehensive evaluation and management.
